# Serological cytokine signature in paediatric patients with inflammatory bowel disease impacts diagnosis

**DOI:** 10.1038/s41598-020-71503-y

**Published:** 2020-09-03

**Authors:** Maiko Tatsuki, Reiko Hatori, Tomoko Nakazawa, Takashi Ishige, Tomoko Hara, Seiichi Kagimoto, Takeshi Tomomasa, Hirokazu Arakawa, Takumi Takizawa

**Affiliations:** 1 Department of Pediatrics, Gunma University Graduate School of Medicine, 3-39-22 Showa-machi, Maebashi, Gunma 371-8511 Japan; 2 Department of Pediatrics, Seikei-Kai Chiba Medical Center, Chiba, Japan; 3 Department of General Pediatrics, Saitama Children’s Medical Center, Saitama, Japan; 4 Pal Children’s Clinic, Isesaki, Japan

**Keywords:** Immunology, Cytokines

## Abstract

Endoscopy is a central tool for diagnosing and evaluating paediatric inflammatory bowel diseases (PIBD), but is too invasive to be frequently repeated in young children. Furthermore, it is challenging to distinguish Crohn’s disease (CD) from ulcerative colitis (UC) endoscopically. This study aimed to determine biomarkers useful for the diagnosis of PIBD. Cytokines, chemokines, and growth factors were quantified in the sera of 15 patients with CD or UC, at disease onset prior to treatment, and 26 age-matched controls. Correlation of cytokine levels with the paediatric CD activity index (PCDAI) and the paediatric UC activity index (PUCAI) was analysed. Interleukin (IL)-6, IL-13, IL-7, and vascular endothelial growth factor were higher in the CD group than in the UC group. The receiver operating characteristic curve analysis showed that IL-7 was a putative biomarker for distinguishing CD from UC (area under the curve: 0.94). Granulocyte–macrophage colony-stimulating factor was associated with PCDAI, and an IL-1 receptor antagonist, IL-6, and macrophage inflammatory protein-1β were associated with PUCAI. These findings indicate significant differences in cytokine signatures among patients with new-onset PIBD, which may improve accuracy in diagnosing PIBD.

## Introduction

An increasing number of children are affected by inflammatory bowel disease (IBD) worldwide, including Crohn’s disease (CD), ulcerative colitis (UC), and unclassified IBD (IBDU)^1–7^. Paediatric IBD (PIBD) is characterised by larger inflammatory lesions^8,9^, more severe phenotypes^10,11^, increased use of immunomodulatory treatment^12^, and longer disease duration than adult-onset IBD.

The Paris classification is the most widely used tool for clinical classification of PIBD and is exclusively based on endoscopic and radiological findings^13–15^. Early diagnosis and detailed assessment are essential for better prognosis in PIBD; nonetheless, it is often difficult to repeat endoscopic procedures in children because they are invasive, particularly for those who require sedation. Additionally, endoscopy can exacerbate symptoms and is not always sufficient for diagnosis. Therefore, non-invasive and easy-to-access diagnostic biomarkers remain an unmet need for PIBD. A body of evidence has shown that cytokines play crucial roles in controlling intestinal inflammation and are associated with the clinical symptoms of IBD^16–18^. In support of this, the randomised REACH study demonstrated the efficacy of infliximab, an anti-tumour necrosis factor-α (TNF-α) chimeric monoclonal antibody, for achieving prolonged response and remission in children with moderate to severe CD^19^. Furthermore, the expression of cytokines, chemokines, and growth factors (hereafter designated as “cytokines”) can be modulated by treatment of the disease using steroids, a key drug for PIBD. Therefore, evaluating their expression levels prior to treatment initiation is essential to pathological understanding and will aid clinicians in diagnosing PIBD. However, few studies have been performed, and little is known about serum cytokines that could distinguish CD from UC in PIBD patients before starting treatment^20,21^.

The present study aimed to determine the cytokine expression profiles in new cases of PIBD and identify putative biomarkers that can be used for diagnosing and evaluating the disease status of PIBD.

## Results

In total, 34 patients were diagnosed with IBD from June 2008 to October 2015. Out of this, 14 and 5 patients were excluded due to early therapeutic interventions and unclear diagnosis, respectively. Therefore, a total of 15 patients underwent further examination. Eight patients with CD (median age, 14.0 years), 7 with UC (median age, 13.0 years), and 26 controls (median age, 11.5 years) were enrolled (Table 1). No differences in age at diagnosis or sex were noted between the CD and UC groups. C-reactive protein (CRP) and erythrocyte sedimentation rate (ESR) were higher in the CD group than in the UC group (*p* < 0.05). The ileocolonic (L3) Paris classification was observed in 75% of CD patients, whereas 85.7% of UC patients had pancolitis (E4). The proportion of CD patients with moderate to severe disease activity on the paediatric Crohn’s disease activity index (PCDAI) (62.5%) was comparable to those with UC (71.4%) on the paediatric ulcerative colitis activity index (PUCAI). Additionally, the simple endoscopic score for CD (SES-CD)^22^ and ulcerative colitis endoscopic index of severity (UCEIS)^23^ were similar between groups (Table 1). The diagnosis of each patient was consistent for more than 5 years after close observation.

**Table 1:** Clinical characteristics of CD, UC, and non-colitis control patients.

Characteristics	CD	UC	Control	*p*-value
Number	8	7	26	
Age, years	14.0 (12.5–15.2)	13.0 (10.5–13.6)	11.5 (9.0–13.5)	n.s.†
Male:female	7:1	2:5	14:12	n.s.‡
**Laboratory data**				
Haemoglobin, g/dL	11.5 (11.0–11.9)	11.8 (10.8–12.9)		n.s
CRP, mg/dL	5.8 (4.5–7.8)	0.8 (0.2–1.7)		< 0.05
ESR, mm/h	68.0 (52.0–77.0)	24.0 (18.0–44.0)		< 0.05
WBC, 1/μL	10,500 (7,250–10,950)	7,500 (6,900–11,150)		n.s
Platelets, × 10^4^/μL	48.0 (44.9–56.1)	34.4 (28.9–42.7)		n.s
FDP, μg/mL	2.3 (2.1–4.7)	1.6 (0.6–2.9)		n.s
D-dimer, μg/mL	0.4 (0.2–0.9)	0.2 (0.1–1.7)		n.s
Albumin, g/dL	2.8 (2.7–3.1)	3.4 (3.3–3.9)		n.s
**Disease classification**				
	L1: 0 (0)	E1: 0 (0)		
	L2: 2 (25.0)	E2: 1 (14.3)		
	L3: 6 (75.0)	E3: 0 (0)		
	L4: 0 (0)	E4: 6 (85.7)		
**Clinical activity**				
**PCDAI:**		**PUCAI:**		
Moderate/severe (> 30)	5 (62.5)	Severe (65–85)	3 (42.8)	
Mild (11–30)	3 (37.5)	Moderate (35–64)	2 (28.6)	
Remission (0–10)	0 (0)	Mild (10–34)	2 (28.6)	
		Remission (0–10)	0 (0)	
**Endoscopic indexes**				
**SES-CD:**		**UCEIS:**		
Severe (≥ 16)	3 (37.5)	Severe (7–8)	2 (28.6)	
Moderate (7–15)	2 (25.0)	Moderate (5–6)	3 (42.8)	
Mild (3–6)	3 (37.5)	Mild (2–4)	2 (28.6)	
Remission (0–2)	0 (0)	Remission (0–1)	0 (0)	

Data are presented as median or proportions.

*ns* not significant, *CD* Crohn’s disease, *UC* ulcerative colitis, *CRP* C-reactive protein, *ESR* erythrocyte sedimentation rate, *WBC* white blood cell count, *FDP* fibrin/fibrinogen degradation products, *PCDAI* paediatric Crohn’s disease activity index, *PUCAI* paediatric ulcerative colitis activity index, *SES-CD* simple endoscopic score for CD, *UCEIS* ulcerative colitis endoscopic index of severity, *L1* terminal ileum, *L2* colonic, *L3* ileocolonic, *L4* upper disease, *E1* ulcerative proctitis, *E2* left-sided UC, *E3* extensive, *E4* pancolitis.

†*p*-value for age from ANOVA test and ‡*p*-value for sex from Fisher’s exact test (across all three groups). Differences between groups were tested using the Mann–Whitney U test for continuous variables.

### Profiles of serum cytokines, chemokines, and growth factors in PIBD patients and controls

**Figure 1:**
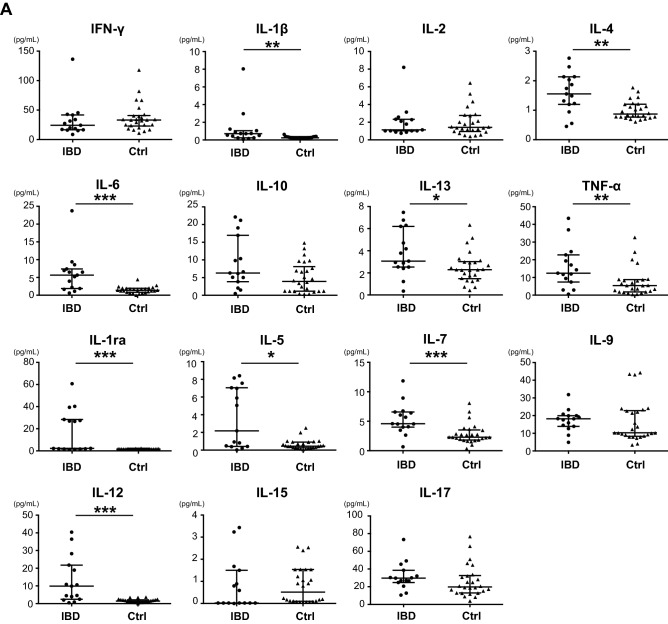

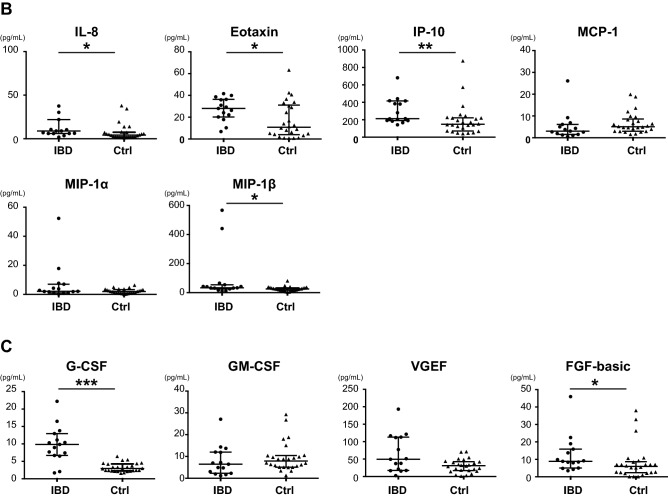
Comparison of serum cytokines **(A)**, chemokines **(B)**, and growth factors **(C)** between IBD and control patients. Mann–Whitney U test was used for comparisons between IBD and control groups. Horizontal lines indicate the median of the group, whereas the top and bottom of the lines represent the 75th and 25th percentiles, respectively. *p < 0.05; **p < 0.01; ***p < 0.001. IBD, n = 15; controls, n = 26.

**Figure 2:**
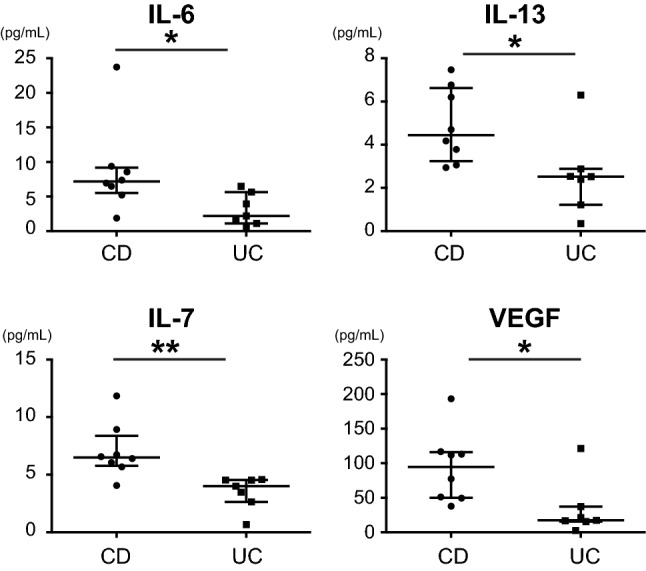
Comparison of serum cytokines, chemokines, and growth factors between CD and UC patients. Post hoc Mann–Whitney U test with Bonferroni correction was used for comparisons between CD and UC groups. Horizontal lines indicate the median of the group, whereas the top and bottom of the lines represent the 75th and 25th percentiles, respectively. *p < 0.05; **p < 0.01. CD, n = 8; UC, n = 7.

We compared the serum cytokine levels between PIBD and control patients. The serum levels of interleukin (IL)-1β, IL-4, IL-6, IL-13, TNF-α, IL-1 receptor antagonist (ra), IL-5, IL-7, IL-12, IL-8, eotaxin, interferon (IFN)-γ-induced protein 10 (IP-10), macrophage inflammatory protein (MIP)-1β, granulocyte–colony-stimulating factor (G-CSF), and fibroblast growth factor (FGF)-basic were significantly higher in the PIBD group than in the control group (Fig. 1). When the CD and UC groups were independently compared to controls, 12 cytokines were higher in the CD group than in the control group, but no cytokines showed higher levels in the UC group (Table 2). Comparison between the CD and UC groups revealed that IL-6, IL-13, IL-7, and VEGF were higher in CD than in UC (Fig. 2 and Table 2).

**Table 2:** Concentrations (pg/mL) of cytokines, chemokines, and growth factors in the serum of CD, UC, and control patients.

		CD	UC	Control	*p*-value*	CD vs. UC†	CD vs. controls†	UC vs. controls†
**Cytokine panel**								
IFN-γ		18.59 (15.14–45.39)	24.37 (8.68–42.78)	33.14 (10.94–117.96)				
IL-1β		0.72 (0.43–8.03)	0.24 (0.06–1.05)	0.25 (0.16–0.62)	< 0.001	n.s	< 0.001	n.s
IL-2		1.79 (1.02–2.58)	1.02 (0.75–3.14)	1.41 (0.39–6.44)				
IL-4		1.86 (1.22–2.76)	1.19 (0.45–2.47)	0.87 (0.60–1.76)	< 0.01	n.s	< 0.001	n.s
IL-6		6.94 (1.88–23.73)	2.20 (0.61–6.48)	1.40 (0.01–4.39)	< 0.001	< 0.05	< 0.001	n.s
IL-10		9.82 (5.03–21.18)	3.84 (0.49–16.95)	3.92 (0.34–14.8)	< 0.05	n.s	< 0.05	n.s
IL-13		4.17 (2.94–7.46)	2.52 (0.35–6.29)	2.28 (0.41–6.32)	< 0.01	< 0.05	< 0.01	n.s
TNF-α		22.76 (9.43–43.45)	7.44 (0.58–17.08)	5.41 (0.39–32.66)	< 0.01	n.s	< 0.001	n.s
IL-1ra		27.4 (1.66–60.7)	1.81 (1.18–40.21)	1.58 (1.18–2.28)	< 0.01	n.s	n.s	n.s
IL-5		6.98 (0.38–8.4)	0.47 (0.02–7.55)	0.44 (0.06–2.5)	< 0.01	n.s	< 0.01	n.s
IL-7		6.4 (4.07–11.84)	4.01 (0.66–4.57)	2.28 (0.35–8.06)	< 0.001	< 0.01	< 0.001	n.s
IL-9		18.89 (13.92–31.83)	15.87 (4.89–19.93)	10.25 (3.41–44.28)				
IL-12		21.77 (3.99–40.41)	2.49 (0.46–10.85)	1.45 (0.06–3.63)	< 0.001	n.s	< 0.001	n.s
IL-15		0.02 (0.02–3.23)	0.59 (0.01–1.5)	0.51 (0.06–2.55)				
IL-17		26.89 (20.59–49.16)	30.05 (10.55–45.49)	19.77 (3.84–76.79)				
**Chemokine panel**								
IL-8		6.95 (3.53–2,175.5)	8.96 (1.83–22.0)	4.19 (1.15–37.95)	< 0.05	n.s	n.s	n.s
Eotaxin		29.32 (17.83–38.69)	26.33 (6.84–41.66)	10.84 (0.73–63.28)				
IP-10		380.67 (183.63–681.66)	205.68 (142.24–418.05)	148.96 (41.0–875.6)	< 0.01	n.s	n.s	n.s
MCP-1		2.72 (1.07–26.07)	3.06 (0.5–9.21)	5.04 (1.55–19.92)				
MIP-1α		4.26 (0.78–52.31)	2.01 (0.17–3.82)	1.96 (0.04–6.33)				
MIP-1β		40.65 (16.92–567.33)	33.31 (10.72–54.02)	25.34 (3.97–81.01)				
**Growth factors**								
G-CSF		10.32 (6.51–16.49)	8.97 (1.68–22.22)	2.94 (1.59–6.44)	< 0.001	n.s	< 0.001	n.s
GM-CSF		4.91 (1.18–13.72)	6.99 (1.67–14.36)	7.91 (1.27–29.27)				
VEGF		77.34 (37.83–193.24)	17.47 (2.55–121.4)	31.1 (1.13–71.31)	< 0.01	< 0.05	< 0.001	n.s
FGF-basic		9.85 (8.02–22.53)	5.0 (3.49–18.93)	5.92 (0.10–38.03)	< 0.05	n.s	< 0.01	n.s

Data are presented as median or numbers (proportions). Differences between the three groups were statistically compared using the Kruskal–Wallis test (*). *P*-value shows the results of Mann–Whitney U test with Bonferroni correction (†).

*ns* not significant, *CD* Crohn’s disease, *UC* ulcerative colitis, *IFN* interferon, *IL* interleukin, *TNF* tumour necrosis factor, *ra* receptor antagonist, *IP-10* IFN-γ-induced protein 10, *MCP* monocyte chemoattractant protein, *MIP* macrophage inflammatory protein, *G-CSF* granulocyte-colony-stimulating factor, *GM-CSF* granulocyte–macrophage colony-stimulating factor, *VEGF* vascular endothelial growth factor, *FGF* fibroblast growth factor.

This result prompted us to perform receiver operating characteristic (ROC) curve analysis to elucidate which factors are superior in distinguishing CD from UC in PIBD patients. The four cytokines with discriminatory power were IL-6 (area under the curve [AUC]: 0.88, 95% CI: 0.70–1.0), IL-13 ([AUC]: 0.89, 95% CI: 0.69–1.0), IL-7 (AUC: 0.95, 95% CI: 0.83–1.0), and VEGF ([AUC]: 0.88, 95% CI: 0.64–1.0). Additional analysis revealed that combinations of these factors did not improve the diagnostic accuracy (Table 3).

**Table 3:** Area under the ROC curves, cut-off value, sensitivity, and specificity in CD and UC patients.

Cytokine	AUC	Sensitivity (%)	Specificity	Cut-off value (pg/mL)	*p*-value
**(A)**					
IL-7	0.94	88	86	5.00	< 0.001
IL-13	0.89	100	86	3.00	< 0.05
VEGF	0.88	100	86	40.00	< 0.05
IL-6	0.88	63	100	6.50	< 0.01
**(B)**					
IL-7, IL-6	0.90	63	100		< 0.001
IL-13, IL-7	0.87	86	88		< 0.001
IL-13, IL-6	0.85	63	100		< 0.001
IL-7, VEGF	0.74	86	88		< 0.05
VEGF, IL-6	0.74	63	100		< 0.05
IL-13, VEGF	0.73	86	88		ns
**(C)**					
IL-13, VEGF, IL-6	0.88	50	86		< 0.001
IL-13, IL-7, IL-6	0.88	50	86		< 0.001
IL-7, VEGF, IL-6	0.76	75	86		< 0.01
IL-13, IL-7, VEGF	0.74	86	88		ns
**(D)**					
IL-13, IL-7, VEGF, IL-6	0.76	84	93		< 0.05

*ROC* receiver operating characteristic, *CD* Crohn’s disease, *UC* ulcerative colitis, *AUC* area under the curve, *IL* interleukin, *VEGF* vascular endothelial growth factor.

Because CRP and ESR levels were significantly different between CD and UC, we thought that the levels of IL-6, IL-13, IL-7, or VEGF might be correlated with CRP or ESR levels. Correlation analysis revealed a moderate correlation, albeit without significance, between CRP levels and each of IL-13 (r = 0.49, *p* = 0.07) and IL-7 (r = 0.48, *p* = 0.08), whereas IL-6 (r = 0.67, *p* < 0.01) and VEGF (r = 0.59, *p* < 0.05) were better correlated with CRP. All the four cytokines, IL-7 (r = 0.25, *p* = 0.38), IL-13 (r = 0.24, *p* = 0.41), VEGF (r = 0.32, *p* = 0.27), and IL-6 (r = 0.54, *p* = 0.05) were moderately correlated with ESR levels, but none of them showed a statistical significance (see Supplementary Fig. S1 online).

### Correlation between endoscopic score, disease activity, and cytokine levels

**Figure 3:**
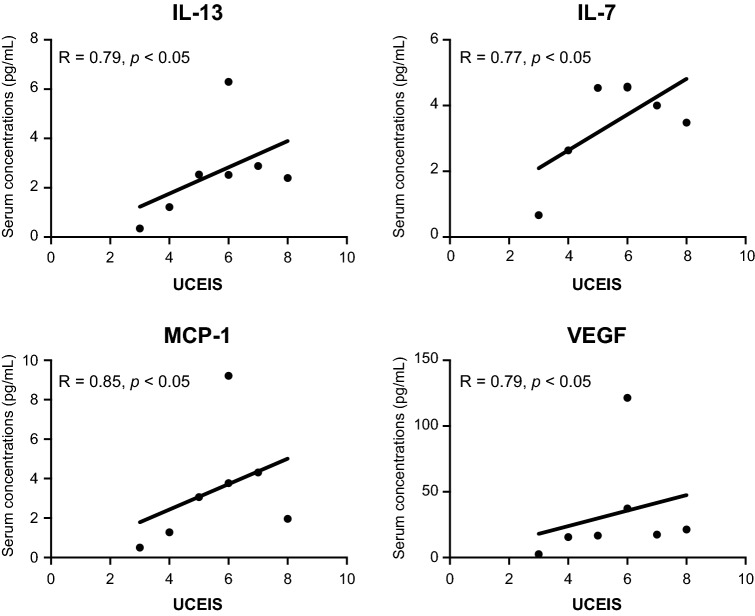
Correlation between the endoscopic scores (UCEIS) and serum cytokine levels. The serum levels of **(A)** IL-13 (r = 0.79, *p* < 0.05), **(B)** IL-7 (r = 0.77, *p* < 0.05), **(C)** MCP-1 (r = 0.85, *p* < 0.05), and **(D)** VEGF (r = 0.79, *p* < 0.05) were strongly correlated with the endoscopic score of UC (UCEIS). Correlation was determined using Spearman’s correlation coefficient.

No correlation between the SES-CD and serum concentrations of any cytokines was found in the CD group; however, in the UC group, the UCEIS showed significant correlation with IL-13 (r = 0.79, *p* < 0.05), IL-7 (r = 0.77, *p* < 0.05), MCP-1 (r = 0.85, *p* < 0.05), and VEGF (r = 0.79, *p* < 0.05) (Table 4, Fig. 3).

**Table 4:** Correlation between the endoscopic scores and serum cytokine levels.

	CD (SES-CD)		UC (UCEIS)	
	Spearman’s r	*p*-value	Spearman’s r	*p*-value
**Cytokine panel**				
IFN-γ	− 0.035	0.946	0.396	0.383
IL-1β	0.227	0.591	0.558	0.204
IL-2	0.319	0.438	0.631	0.141
IL-4	0.383	0.345	0.558	0.204
IL-6	− 0.179	0.675	0.378	0.406
IL-10	− 0.071	0.871	0.684	0.1
IL-13	− 0.203	0.63	0.792	< 0.05
TNF-α	0.538	0.178	0.451	0.318
IL-1ra	0.371	0.363	0.378	0.406
IL-5	0.455	0.258	0.558	0.204
IL-7	0.347	0.399	0.774	< 0.05
IL-9	0.203	0.63	0.486	0.277
IL-12	− 0.072	0.872	0.414	0.359
IL-15	− 0.274	0.511	0.272	0.548
IL-17	0.503	0.209	0.631	0.141
**Chemokine panel**				
IL-8	− 0.071	0.871	0.541	0.221
Eotaxin	0.467	0.247	− 0.018	0.985
IP-10	0.263	0.523	0.072	0.883
MCP-1	− 0.131	0.756	0.846	< 0.05
MIP-1α	0.395	0.339	0.054	0.919
MIP-1β	0.215	0.609	0.505	0.257
**Growth factors**				
G-CSF	− 0.012	0.988	0.684	0.1
GM-CSF	− 0.108	0.808	0.738	0.066
VEGF	− 0.574	0.143	0.792	< 0.05
FGF-basic	0.119	0.779	0.576	0.187

*CD* Crohn’s disease, *SES-CD* simple endoscopic score for CD, *UC* ulcerative colitis, *UCEIS* ulcerative colitis endoscopic index of severity, *IFN* interferon, *IL* interleukin, *TNF* tumour necrosis factor, *ra* receptor antagonist, *IP-10* IFN-γ-induced protein 10, *MCP* monocyte chemoattractant protein, *MIP* macrophage inflammatory protein, *G-CSF* granulocyte-colony-stimulating factor, *GM-CSF* granulocyte–macrophage colony-stimulating factor, *VEGF* vascular endothelial growth factor, *FGF* fibroblast growth factor.

Multivariable linear regression analysis, with the parameters of all 25 cytokines, revealed a model indicating that granulocyte–macrophage colony-stimulating factor (GM-CSF) was correlated with PCDAI (R^2^ = 0.51, F = 6.23, *p* = 0.04) (Table 5). IL-1ra, IL-6, and MIP-1β were correlated with PUCAI (R^2^ = 0.99, F = 94.47, *p* < 0.01) in the UC group (Table 5).

**Table 5:** Summary of multiple linear regression for disease activity of CD and UC.

Disease	Variable	B	SEB	Β
CD	GM-CSF	1.83	0.73	0.71
UC	IL-1ra	− 1.79	0.15	− 1.05
	IL-6	4.85	0.78	0.40
	MIP-1β	0.61	0.15	0.37

*B* un-standardised regression coefficients, *SEB* standard error of B, *β* standardised regression coefficients, *CD* Crohn’s disease, *UC* ulcerative colitis, *GM-CSF* granulocyte–macrophage colony-stimulating factor, *IL* interleukin, *ra* receptor antagonist, *MIP* macrophage inflammatory protein.

## Discussion

Our results indicated that cytokines were differentially expressed in the sera of CD and UC patients prior to treatment, suggesting that these biomarkers can aid in the diagnosis of PIBD. We observed 12 differentially expressed cytokines in the new CD cases, but none in the new UC cases when compared to controls. Furthermore, we identified IL-7 as a candidate biomarker for distinguishing CD from UC. There were no factors associated with endoscopic scoring in CD, whereas 4 factors were associated with UC—namely, IL-13, IL-7, MCP-1, and VEGF. GM-CSF, IL-1ra, IL-6, and MIP-1β were correlated with PCDAI in CD and PUCAI in UC.

A previous study attempting to identify serum cytokine profiles to differentiate CD and UC in adult patients reported that IFN-γ, IL-6, and IL-7 were higher in CD and growth-regulated oncogene and TNF-α were higher in UC than in controls, but no cytokines were differentially expressed between CD and UC^24^. The study used a generalised linear model to discriminate UC and CD, including age, sex, and 15 cytokines as parameters and observed an AUC of 0.936^24^. The current study observed clear differences in cytokine profiles between CD and UC, with IL-7 alone having the highest diagnostic accuracy. The discrepancy in results between the previous and current studies might be attributed to the age and treatment of participants. As mentioned earlier, PIBD has different clinical presentation than adult IBD, which suggest differences in underlying pathology. The distinct cytokine expression profiles observed in this study, especially in CD patients, likely reflect the differences in pathology between adults and children.

The use of medication was also different between our current study and others. No participants in this study received IBD treatment, such as 5-aminosalicylic acid, steroids, immunomodulators, biologics, antibiotics, blood cell apheresis, or surgery, prior to sample collection. These treatments can decrease gastrointestinal inflammation and affect serum cytokine expression profiles. It is vital to determine baseline cytokine profiles of IBD patients to determine their utility as diagnostic biomarkers, but most studies on serum cytokines in PIBD do not indicate whether or not participants underwent treatment. Fujitake et al. investigated IL-4, IL-5, IL-6, IFN‐γ, TNF‐α, and TGF‐β1 in CD patients who were not taking medication and reported that serum IL-6 and TNF-α were higher in the acute phase than in the remission phase^20^. Kader et al. reported that the placental growth factor, IL-7, TGF‐β1, and IL-12p40, were higher in the active phase of CD than in the remission phase^25^. We found 12 cytokines that were increased in the active CD at disease onset. We did not compare the active and remission phases, but rather diseases and controls. The larger number of differentially expressed cytokines in this study may be attributable to this difference in comparison.

IL-7 had the highest diagnostic accuracy to distinguish CD from UC. IL-7 was shown to be required for T-cell development and is secreted from stromal and epithelial cells^26^. It is produced by intestinal epithelial cells, and its overexpression induces spontaneous development of chronic colitis in mice^27^. Furthermore, the IL-7 receptor has been proposed as a therapeutic target for IBDs^28,29^. These previous studies strongly demonstrate that IL-7 is involved in the pathology of IBD. However, there is a report showing a contradictory result, in which serum IL-7 levels were lower among patients at diagnosis and in the remission and active phases than among controls^30^. These results indicate that serum IL-7 levels are likely dependent on the patient’s condition. We found that IL-7 was significantly higher in new paediatric cases of CD than in those of UC. Further studies are required to elucidate whether IL-7 can serve as a biomarker to distinguish CD from UC in PIBD patients.

We found that CRP and ESR were significantly higher in the CD group than in the UC group. Consistently, Fagan et al.^31^ reported elevation of CRP and ESR in both CD and UC patients, with higher elevation in CD than in UC for all categories of disease severity. Interestingly, Torres et al. recently reported that serum antibodies and proteins change 5 years before CD, but not at UC onset in adult patients^32^. Although this previous study differs from ours in terms of the age of participants and timing of measurement, they both indicate that inflammation in CD is intensive and distinct from that in UC. Collectively, our results indicated that more cytokines were upregulated in CD than in UC, and that the extent of systemic inflammation might be higher in CD than in UC among PIBD patients.

A recent study of children with CD demonstrated the lack of correlation among the clinical disease activity index, the PCDAI, and endoscopic severity^33^. Consistent with this, we did not observe a significant correlation between disease activity and SES-CD. A position paper by the European Society for Paediatric Gastroenterology, Hepatology, and Nutrition does not recommend correlating endoscopic scores and clinical symptoms in UC patients^34^. In accordance with this, there was no significant correlation between PUCAI and UCEIS in this study.

We found that GM-CSF, a potent growth and differentiation factor in myeloid cells, was correlated with PCDAI. Myeloid cells are responsible for the anti-microbial activity, and neutralisation of GM-CSF using anti-GM-CSF autoantibodies is proposed to be associated with disease progression and relapse of IBD^16,17^. These results suggest that GM-CSF may be a potential marker of disease activity in new-onset paediatric CD patients. PUCAI was correlated with a model including IL-1ra, MIP-1β, and IL-6, although serum levels of these cytokines were not significantly different between the UC group and controls. This indicates that these factors may reflect disease activity, but not morbidity. IL-6 has been reported to be associated with steroid resistance and disease activity in paediatric patients with severe UC^18^ and may be a putative marker of disease activity in paediatric UC patients. In adult patients, reduction of serum IL-6 measured within a couple of months after beginning therapy with biologics can be used to predict the effects of the biologics 12 months after their use to treat both CD and UC, indicating that IL-6 levels correlate with disease activity in both children and adults^35^.

A limitation of this study was the small number of subjects from a single institution. An increase in the number of patients would result in more uniform groups and potentially reveal differences among PIBD subtypes with different immunopathogeneses. Further, the extent of inflammation differed between UC and CD in this study. Because it was unclear how this difference affects the serum cytokine levels, we tested the correlation of cytokine levels with CRP or ESR (see Supplementary Fig. S1 online). There was a moderate correlation between CRP and each of IL-6 and VEGF, supporting the specific increase in IL-7 in CD patients in the current study. However, the number of participants was small, and thus, future studies should investigate whether and how active inflammation in CD with high ESR and CRP levels affect serum cytokine levels. The current results thus need to be confirmed in a larger patient population from multiple institutes before they can be applied in clinical settings.

In conclusion, endoscopic or pathological findings in new-onset PIBD are different from those in adults. Rothschild et al.^36^ reported that granulomas were only found in 34% of paediatric CD patients. Additionally, crypt abscesses, which are the basis for the diagnosis of UC, are a nonspecific manifestation of several forms of enteritis. An epidemiological survey estimated that the morbidity rate of IBDU was approximately 15% in children and 5% in adults^37^. Therefore, easily accessible biomarkers for diagnosing new-onset PIBD are clinically relevant. The serological features of several cytokines allow for disease identification. Finally, we found that serum IL-7 was a useful non-invasive marker for distinguishing CD from UC in new cases of PIBD.

## Methods

### Study population and sample collection

We enrolled consecutive patients who had been diagnosed with IBD, either CD or UC based on Porto criteria^38^, at the Department of Pediatrics of Gunma University Hospital or Saitama Children’s Medical Center in Japan from June 2008 to October 2015. Consequently, CD and UC diagnoses were established based on standard clinical, endoscopic, and histological criteria (Table S1). Endoscopy was performed at the same time of serum collection. The inclusion criteria for patients included: active stage of disease, but no prior treatment at the time of serum sampling. Exclusion criteria included: IBDU diagnosis, history of autoimmune diseases, or previous treatment (5-aminosalicylic acid, steroids, immunomodulators, biologics, antibiotics, blood cell apheresis, or surgery) at the time of serum sampling. Control samples were obtained from age-matched patients with chronic functional constipation but without IBD or any other inflammatory disorders.

### Collection of clinical information

Clinical characteristics including age at diagnosis, sex, laboratory findings (haemoglobin, CRP, ESR, white blood cell count, thrombocytes, fibrin/fibrinogen degradation products, D-dimer, and serum albumin), disease types, clinical activity, endoscopic findings, and treatment received were obtained from medical records. Disease location was based on the Paris classification: L1 (terminal ileum), L2 (colonic), L3 (ileocolonic), and L4 (upper disease) for CD and E1 (ulcerative proctitis), E2 (left-sided UC), E3 (extensive), and E4 (pancolitis) for UC^15,39^. Disease activity was assessed using the PCDAI^40^ or PUCAI^41^. The endoscopic index was graded according to SES-CD^22^ or UCEIS^23^. The SES-CD assesses mucosal ulcer size, ulcerated surface, endoscopic extension, and presence of stenosis^22^. The SES-CD was stratified into four categories: remission (SES-CD 0–2), mild (SES-CD 3–6), moderate (SES-CD 7–15), and severe (SES-CD > 15). The UCEIS consists of visible vascular patterns, bleeding, erosions, and ulcers. The UCEIS was stratified into four categories: remission (UCEIS 0–1), mild (UCEIS 2–4), moderate (UCEIS 5–6), and severe (UCEIS 7–8).

### Cytokine measurement

Serum concentrations of 25 humoral factors including 15 cytokines (IFN-γ, IL-1β, IL-2, IL-4, IL-6, IL-10, IL-13, TNF-α, IL-1ra, IL-5, IL-7, IL-9, IL-12, IL-15, IL-17), 6 chemokines (IL-8, eotaxin, IP-10, MCP-1, MIP-1α, MIP-1β), and four growth factors (G-CSF, GM-CSF, VEGF, FGF-basic) were determined using a double-beam laser automatic analyser (Bio-Plex Protein Assay System; Bio-Rad, USA). Serum samples were frozen at − 80 °C immediately after collection and were stored until analysis. All samples were thawed on ice, vortexed, centrifuged at 14,000 × *g* for 10 min at 4 °C, and diluted with the standard diluent in the kit (Bio-Plex Pro Human Cytokine 27-Plex Assay #M500KCAF0Y; ratio 1:4 by volume). Each sample was measured more than twice. The mean values of measurements were used as the representative values for each subject.

### Statistical analyses

Patient characteristics are presented as median and interquartile range or as proportions. Differences between groups were determined using the Mann–Whitney U test, Student’s t-test, or Fisher’s exact test. Serum cytokine concentrations are presented as mean ± SEM from three independent measurements. Differences between the three groups (CD, UC, and control groups) were statistically compared using the Kruskal–Wallis test, and post hoc Mann–Whitney U test with Bonferroni correction was used for comparisons between two of the three groups. A ROC curve was used to determine the appropriate cut-off cytokine levels for distinguishing CD from UC. Correlations between the endoscopic scores (SES-CD, UCEIS) and serum cytokine levels were established using nonparametric Spearman’s correlation. Multiple linear regression analysis was used to predict disease activity (PCDAI, PUCAI) based on cytokines.

Statistical analyses were performed using JMP14 (SAS Institute Inc., Cary, NC, USA) and GraphPad Prism version 7.00 for Windows (GraphPad Software, La Jolla, CA, USA), and *p*-values < 0.05 were considered statistically significant.

### Ethical considerations

This study was approved by the Medical Ethical Committee of the Gunma University Hospital (protocol number HS2019-018) and Saitama Children’s Medical Center (protocol number 2019-05-024) and was conducted in accordance with all ethical principles of the Seventh Revision of the Helsinki Declaration from 2013. Samples were initially collected for another study, namely "Epigenetics of Inflammatory Bowel Disease" (protocol number 107: approved by the Medical Ethical Committee of the Gunma University Hospital). Serum samples were obtained after patients, and their parents/guardians provided informed consent, and participation in the study was voluntary. All experiments were performed in accordance with the approved guidelines.

## Supplementary information

Supplementary Information.

Supplementary Figure 1.

## Data Availability

The datasets generated and analysed during this study are available from the corresponding author upon reasonable request.
