# Supplementary material for: Serological cytokine signature in paediatric patients with inflammatory bowel disease impacts diagnosis
Source: Sci Rep. 2020 Sep 3;10:14638. doi: 10.1038/s41598-020-71503-y (PMC7471680; doi:10.1038/s41598-020-71503-y)
Supplement: Supplementary file 2 — Supplementary Figure 1. [file 41598_2020_71503_MOESM2_ESM.docx]

**Supporting Information**

**Serological Cytokine Signature in Paediatric Patients with Inflammatory Bowel Disease Impacts Diagnosis**

Maiko Tatsuki^1^, Reiko Hatori^1^, Tomoko Nakazawa^2^, Takashi Ishige^1^, Tomoko Hara^3^, Seiichi Kagimoto^3^, Takeshi Tomomasa^4^, Hirokazu Arakawa^1^, Takumi Takizawa^1*^

**
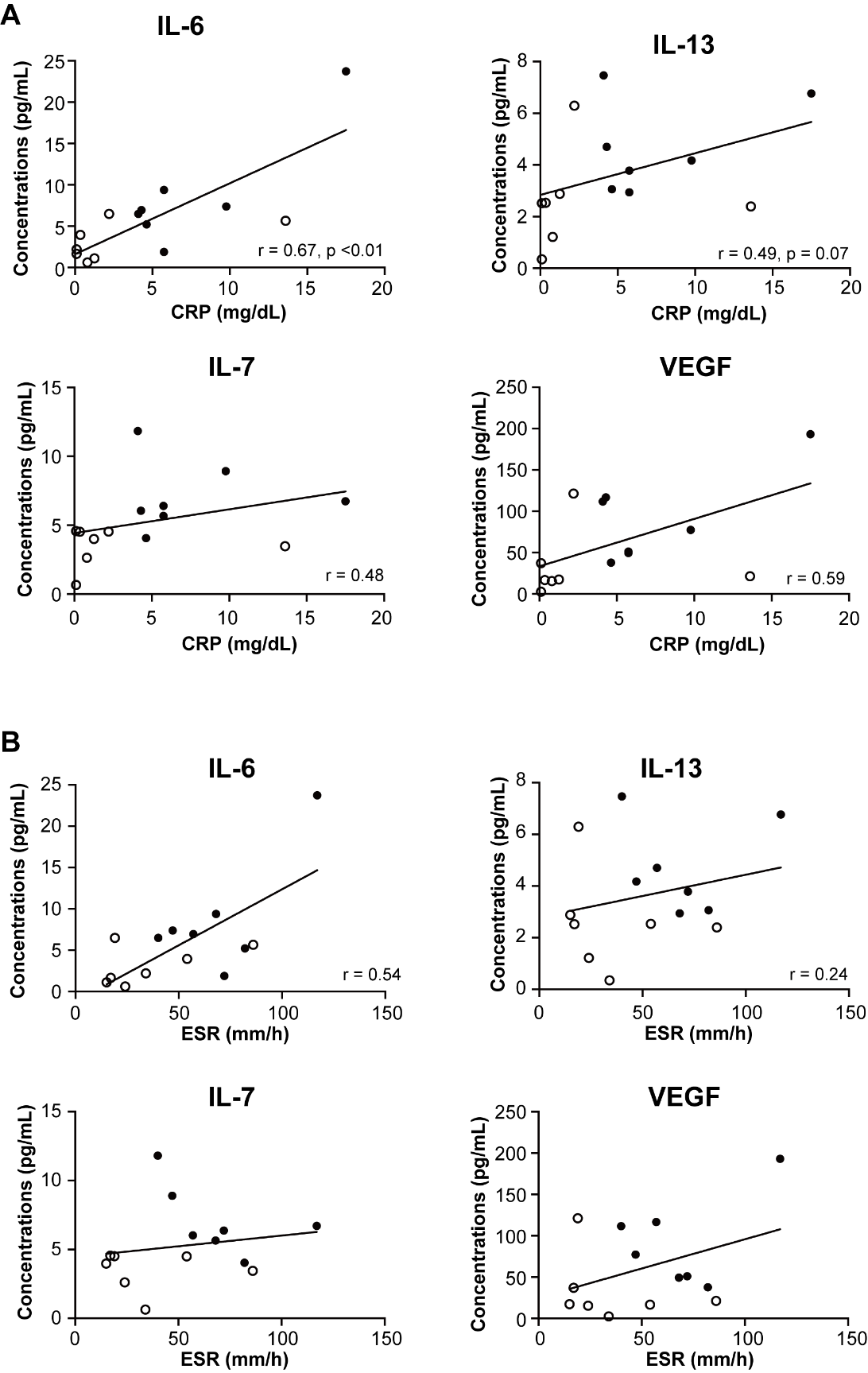
**

**Supplementary Figure S1.** Correlations among CRP, ESR, and serum levels of IL-6, IL-13, IL-7, and VEGF. Comparison of cytokines and CRP (A) or ESR (B). Correlation was tested with Spearman’s rank correlation. Closed dots indicate Crohn’s disease (CD) and open dots indicate ulcerative colitis (UC).
